# Prevalence of Antibiotic-Resistant *Shigella* spp. in Bangladesh: A Systematic Review and Meta-Analysis of 44,519 Samples

**DOI:** 10.3390/antibiotics12050817

**Published:** 2023-04-26

**Authors:** Saleh Ahmed, Md Imrul Hasan Chowdhury, Shabiha Sultana, Sayeda Sadia Alam, Mahfuza Marzan, Md Asiful Islam

**Affiliations:** 1 Center for Biotechnology and Genomic Medicine, Medical College of Georgia, Augusta University, Augusta, GA 30912, USA; salahmed@augusta.edu; 2 Department of Biochemistry and Molecular Biology, The University of Texas Medical Branch, Galveston, TX 77555, USA; mdchowdh@utmb.edu; 3 Department of Cellular Biology and Anatomy, Medical College of Georgia, Augusta University, Augusta, GA 30912, USA; ssultana@augusta.edu; 4 Department of Biochemistry and Molecular Biology, Jahangirnagar University, Savar, Dhaka 1342, Bangladesh; sadiacx70@gmail.com; 5 Department of Microbiology, Jahangirnagar University, Savar, Dhaka 1342, Bangladesh; mmarzan@juniv.edu; 6 Department of Chemistry, University of South Florida, 4202 E Fowler Ave, Tampa, FL 33620, USA; 7 WHO Collaborating Centre for Global Women’s Health, Institute of Metabolism and Systems Research, College of Medical and Dental Sciences, University of Birmingham, Birmingham B15 2TT, UK

**Keywords:** *Shigella* spp., shigellosis, antimicrobial resistance, prevalence, systematic review, meta-analysis, Bangladesh

## Abstract

Shigella is the leading global etiological agent of shigellosis, especially in poor and underdeveloped or developing nations with insufficient sanitation such as Bangladesh. Antibiotics are the only treatment option for the shigellosis caused by *Shigella* spp. as no effective vaccine exists. However, the emergence of antimicrobial resistance (AMR) poses a serious global public health concern. Therefore, a systematic review and meta-analysis were conducted to establish the overall drug resistance pattern against *Shigella* spp. in Bangladesh. The databases of PubMed, Web of Science, Scopus, and Google Scholar were searched for relevant studies. This investigation comprised 28 studies with 44,519 samples. Forest and funnel plots showed any-drug, mono-drug, and multi-drug resistance. Any fluoroquinolone had a resistance rate of 61.9% (95% CI: 45.7–83.8%), any trimethoprim–sulfamethoxazole—60.8% (95% CI: 52.4–70.5%), any azithromycin—38.8% (95% CI: 19.6–76.9%), any nalidixic acid—36.2% (95% CI: 14.2–92.4%), any ampicillin—34.5% (95% CI: 25.0–47.8%), and any ciprofloxacin—31.1% (95% CI: 11.9–81.3%). Multi-drug-resistant *Shigella* spp. exhibited a prevalence of 33.4% (95% CI: 17.3–64.5%), compared to 2.6% to 3.8% for mono-drug-resistant strains. Since resistance to commonly used antibiotics and multidrug resistance were higher, a judicious use of antibiotics, the promotion of infection control measures, and the implementation of antimicrobial surveillance and monitoring programs are required to tackle the therapeutic challenges of shigellosis.

## 1. Introduction

*Shigella* is a rod-shaped, Gram-negative, and non-spore-forming bacterium that was named after Kiyoshi Shiga who discovered it in 1897. As a facultative anaerobic and nonmotile bacterium, *Shigella* is closely genetically related to *E. coli* [1]. Biochemical reactions, serological differences, and genetic relatedness categorize *Shigella* into four species: *S*. *dysenteriae*, *S. flexneri*, *S. boydii*, and *S*. *sonnei* [2]. It is considered the sole causative agent of shigellosis, a highly contagious intestinal infection. The clinical symptoms of shigellosis usually manifest from 12 h to 3 days after exposure, and include bloody diarrhea, a high fever, abdominal pain, and tenesmus [3,4,5,6]. Self-recovery occurs after 5 to 7 days from the onset of symptoms. Shigellosis has a very high frequency in developing countries because of poor hygiene and sanitation and food contamination. The most prevalent species found in developing countries are *S. dysenteriae, S. flexneri*, and *S. boydii* [7,8]. Children younger than 5 years old are the most likely to be infected with *Shigella*, but individuals of all ages can be infected. As it is highly contagious, *Shigella* can spread from tiny particles of infected stools, and from person-to-person via interpersonal contact.

Approximately 700 million diarrheal diseases contributed to the global burden in 2015, with 500,000 accompanying deaths [9]. As a diarrheal disease, shigellosis could be a greater threat in the near future [10]. The treatment plan of shigellosis is totally dependent on the disease’s severity. In the case of mild symptoms, treatment is focused mainly on hydration and electrolyte management because of the excessive loss of water. In most severe cases of shigellosis, and for immunocompromised individuals, antibiotics are prescribed to treat the infection. Prior knowledge of drug susceptibility is a requirement before choosing a treatment plan for the affected individual. However, the repetitive and inappropriate use of antibiotics to treat shigellosis may contribute to antibiotic resistance. The incorrect use of antibiotics may also worsen the symptoms and lead to fatalities [11]. Antibiotic resistance has become an alarming global issue in recent years. In 2016, the World Health Organization (WHO) reported the *Shigella* species to be one of eight threatening bacteria exhibiting drug resistance [12]. The clinical management of shigellosis is globally becoming more challenging, especially in developing countries [13]. Drug choices for treating *Shigella*-spp.-mediated diarrhea include fluoroquinolones, cephalosporins, and sulfonamides [14]. However, the bacteria are becoming increasingly more resistant to maximal antibiotics through different drug resistance mechanisms, thereby limiting the treatment scope of drug treatments [15,16,17]. *Shigella* spp. develops drug resistance by decreasing outer-membrane permeability, extruding drugs with an active efflux system, increasing the expression of drug-inactivating and -modifying enzymes, and with target modification with mutations [17,18,19].

The drug resistance pattern of a country usually represents the effectiveness of the current treatment plan of any disease and sets the future direction. To battle the global burden and limit the wide spread of antimicrobial resistance, regular surveillance of the drug resistance of shigellosis should be highly considered. As a developing country, Bangladesh has a high prevalence of shigellosis, and an antibiotic resistance pattern was observed in different published studies because of the injudicious use of antibiotics. However, these data reflect region-specific information, and no systematic review has been conducted to show the drug resistance pattern of shigellosis in Bangladesh. Therefore, this systematic review and meta-analysis (SRMA) represent an approach for the updated and comprehensive assessment of the shigellosis drug resistance burden in Bangladesh.

## 2. Results

### 2.1. Study Selection

**Figure 1:**
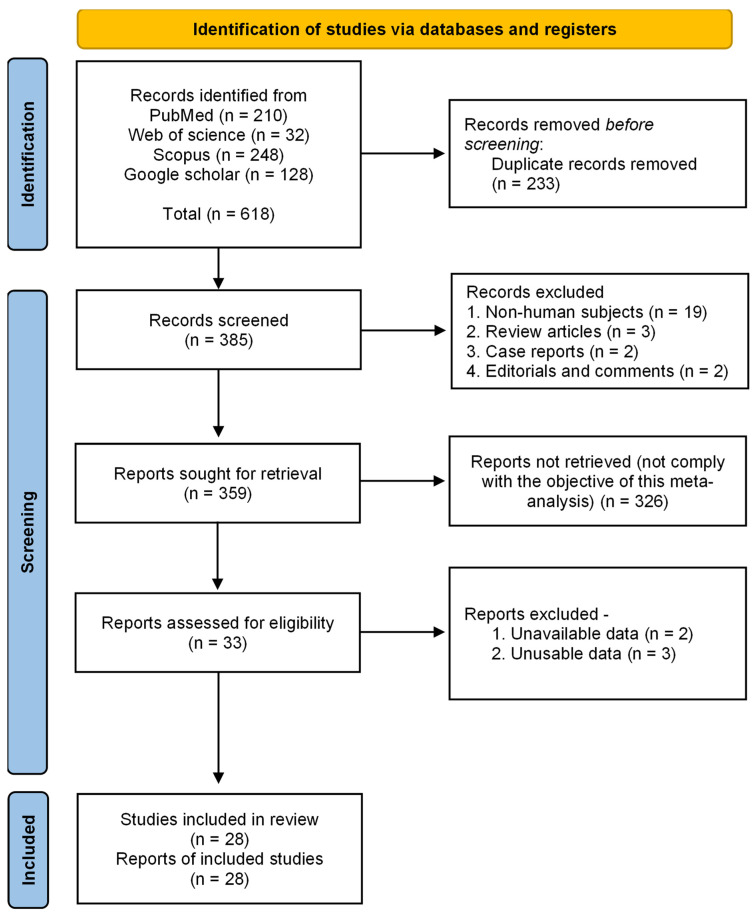
PRISMA flow diagram showing study selection process.

We initially retrieved 618 studies based on the results of the conducted searches across 4 databases: PubMed, Web of Science, Scopus, and Google Scholar. After the initial eligibility check for prescreening, and the removal of duplicate records (233), the titles and abstracts of 385 records were examined. After the initial screening, a total of 26 studies (19 nonhuman studies, 3 review articles, 2 case reports, and 2 editorials) were excluded from the study. The 359 remaining studies were sought for retrieval, of which 326 were eliminated for failing to meet the objective of this systematic review and meta-analysis (SRMA), and 33 studies were retained for the assessment of their full text for eligibility. Lastly, 28 studies were included to conduct this SRMA after the removal of 5 incomplete studies with unavailable data (Figure 1).

### 2.2. Characteristics of the Included Studies

In this SRMA, a total of 44,519 samples collected from 6 different areas (Dhaka, Kushtia, Rajbari, Mirzapur, Matlab, and Teknaf) of Bangladesh and examined for the presence of anti-microbial resistance were included. The majority of the included studies (>80%) were conducted in Dhaka city of Bangladesh. For the antimicrobial susceptibility test (AST), the disk diffusion method was used in most of the studies. The ages of the participants reported in the study ranged between ≤5 years and ≥60 years. Regarding the severity of disease, only 4 out of 28 included studies reported severity of dehydration: no dehydration, moderate or some dehydration, and severe dehydration [3,20,21,22]. The major characteristics of the 28 included studies with references are listed in Table 1.

### 2.3. Quality Assessment

**Figure 2:**
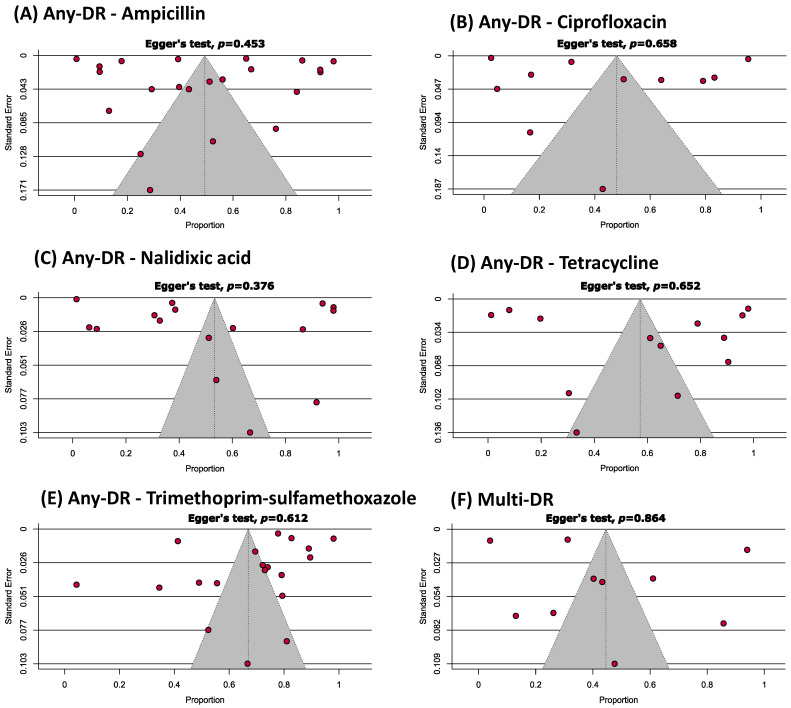
Funnel plots estimating the prevalence of any-drug (**A**–**E**) and multi-drug (**F**) resistances representing an absence of publication bias.

In the Supplemental Materials, Tables S2 and S3 provide a comprehensive quality assessment of the included studies (cross-sectional and cohort) based on the JBI critical appraisal tools. It was determined that 50% of the publications were of a high quality with a low risk of bias and 42.9% were of a moderate quality (with a moderate risk of bias). Egger’s test results and a visual examination of the funnel plot revealed that no analysis displayed a single publication bias (Figure 2).

### 2.4. Overall Antibiotic Resistance Pattern

**Figure 3:**
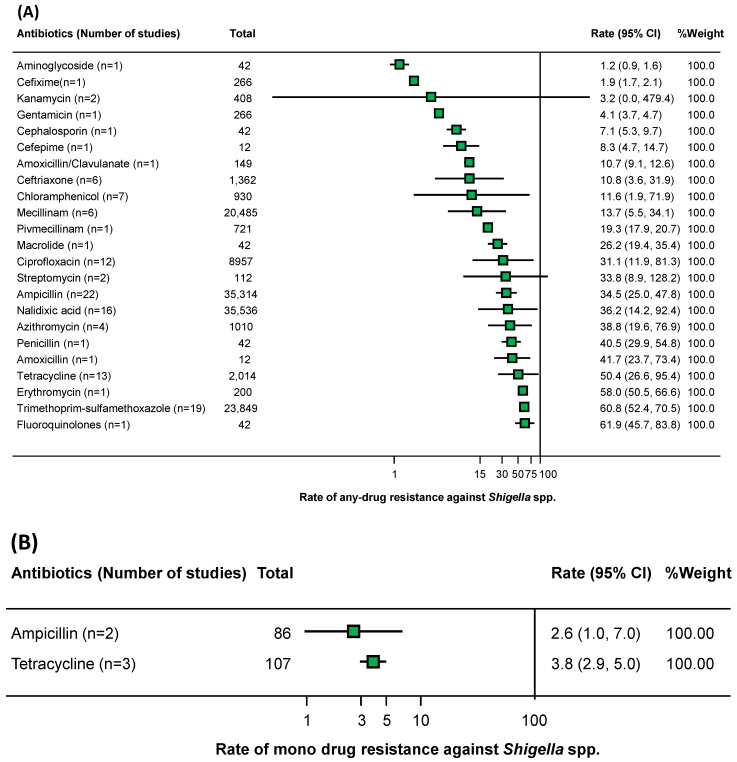

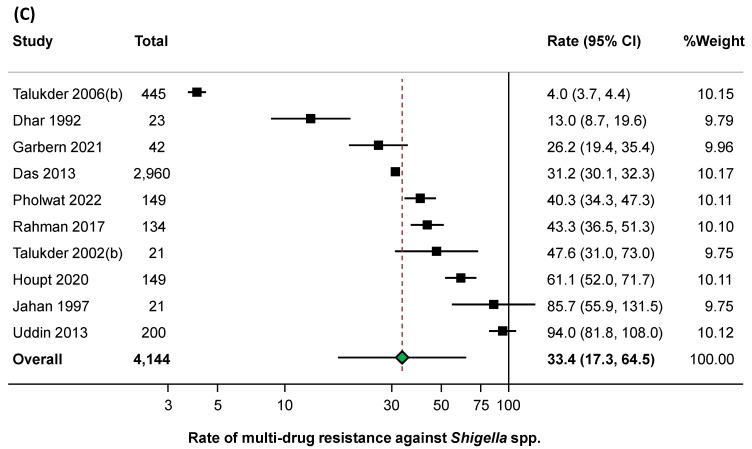
Forest plots estimating the prevalence of any-drug (**A**), mono-drug (**B**), and multi-drug (**C**) resistances against *Shigella* spp. [20,21,24,26,28,30,34,38,41,43].

Forest plots were developed to determine the prevalence of any-drug, mono-drug, and multi-drug resistance in shigellosis patients from Bangladesh (Figure 3). The meta-analysis of any-drug resistance showed that the highest prevalence rate was found in any-fluoroquinolones, at 61.9% (95% CI: 45.7–83.8%), while the lowest prevalence rate was in any-aminoglycosides, at 1.2% (95% CI: 0.9–1.6%). Considering the higher number of tested samples (*n* > 800), the significant resistance rate was observed in any-trimethoprim–sulfamethoxazole at 60.8% (95% CI: 52.4–70.5%), any-tetracycline at 50.4% (95% CI: 26.6–95.4%), any-azithromycin at 38.8% (95% CI: 19.6–76.9%), any-nalidixic acid at 36.2% (95% CI: 14.2–92.4%), any-ampicillin at 34.5% (95% CI: 25.0–47.8%), any-ciprofloxacin at 31.1% (95% CI: 11.9–81.3%), any-mecillinam at 13.7% (95% CI: 5.5–34.1%), any-chloramphenicol at 11.6% (95% CI: 1.9–71.9%), and any-ceftriaxone at 10.8% (95% CI: 3.6–31.9%). (Multi-drug-resistant *Shigella* spp. had an overall prevalence of 33.4% (95% CI: 17.3–64.5%), while mono-resistant had a prevalence of 2.6% (95% CI: 1.0–7.0%) to 3.8% (95% CI: 2.9–5.0%).

### 2.5. Representation of Outlier Studies with Prevalence of Any-Drug and Multi-Drug Resistance

**Figure 4:**
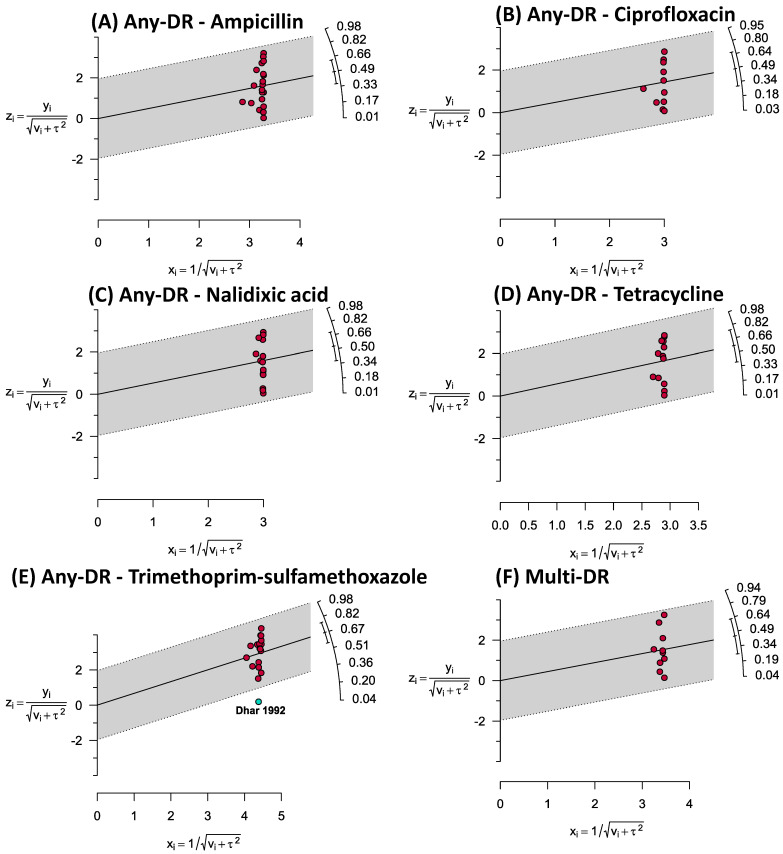
Galbraith plots representing the outlier studies estimating the prevalence of any-drug (**A**–**E**) and multi-drug (**F**) resistances.

Using a higher number of studies (*n* ≥ 12), Galbraith plots were created to represent outlier studies measuring the prevalence of any-drug (any-ampicillin, any-ciprofloxacin, any-nalidixic acid, any-tetracycline, and any-trimethoprim–sulfamethoxazole) and multi-drug resistances (Figure 4). Only one study (Dhar 1982) was identified to be an outlier in our analysis.

### 2.6. Subgroup and Sensitivity Analysis

Subgroup analysis of adult and child combined vs. only child showed that the only child had a higher prevalence of any tested drug resistance against *Shigella* spp. compared to child and adult combinedly. The prevalence rate of mecillinam resistance was found to be highest in the child alone at 93.7% (95% CI: 89.7–97.7%), followed by trimethoprim–sulfamethoxazole at 74% (95% CI: 65.0–84.3%), ciprofloxacin at 62.3% (95% CI: 64.0–107.9%), ampicillin at 43.0% (95% CI: 25.6–72.4%), and ceftriaxone at 32.4% (95% CI: 30.0–35.1%) (Figure S1). By excluding small (*n* < 100), low-quality, and moderate-quality studies, sensitivity analyses were carried out to measure the prevalence of any tested antibiotic and multi-drug resistance. The results revealed minor variations in the re-estimated overall prevalence (Table 2). Overall, no study has considerably changed the pooled prevalence of any-drug- and multi-drug-resistant *Shigella* spp., which indicates that our findings are robust and trustworthy.

## 3. Discussion

Antimicrobial resistance is becoming a growing concern in public health due to the prevalence of infectious diseases; it now appears to be an alarming issue globally. Antimicrobial resistance poses a significant challenge as it limits the effectiveness of existing treatments and increases the risk of treatment failure. If the antibiotic resistance issue is left unaddressed, a single person will die every 3 s, which is projected to cause 10 million deaths by 2050 [47]. Therefore, there is an urgent need to develop new antimicrobial agents and alternative treatment strategies to combat this issue.

The therapeutic management of *Shigella* spp. is becoming more challenging due to the emergence of antimicrobial-resistant *Shigella* strains. A change in the selection of antibiotics has been required over the years because of this resistance issue. A global enteric multicenter study (GEMS) previously found that improper hygiene practices, the uncovering of a water storage container, irregular hand washing practice, and no usages of hand washing substances are the major contributing causes to shigellosis. The children of low-income families are the most susceptible to shigellosis in childhood [48,49]. Bangladesh, with limited resources, is not an exception to this case. In this systematic review and meta-analysis (SRMA), the prevalence of antibiotic resistance against *Shigella* spp. in Bangladesh is addressed, which consists of 28 relevant studies conducted up until 11 March 2023. So, this study carries a remarkable significance for a better understanding of the antimicrobial resistance pattern of *Shigella* spp. in Bangladesh and will be useful for launching future policies and treatment strategies. According to our meta-analysis, any-fluoroquinolones showed the highest resistance rate of 61.9% (95% CI: 45.7–83.8%); the lowest resistance rate of 1.2% (95% CI: 0.9–1.6%) was shown by any-aminoglycosides. The rate of ampicillin resistance is 36.2% (95% CI: 14.2–92.4%), which is far below that of Ethiopia at a rate of 83.1% (95% CI 75.7–88.6) (4). Similar is true for amoxicillin, which bears a resistance rate in Bangladesh of 41.7% (95% CI: 23.7–73.4%) and in Ethiopia of 84.1% (95% CI 75.6–90.1). However, the ciprofloxacin resistance rate is found to be uplifted in Bangladesh at 31.1% (95% CI: 11.9–81.3%), whereas in Ethiopia it is 8.9% (95% CI 6.0–12.8) [15]. Bangladesh is also ahead of Ethiopia in ceftriaxone resistance, with a rate of 10.8% (95% CI: 3.6–31.9).

However, MDR *Shigella* spp. has an overall prevalence of 33.4% (95% CI: 17.3–64.5%) in Bangladesh, which was found to be lower than that of Ethiopia at 83.2% (95% CI 77.1–87.9) [15]. This decrement could be the result of the improvement of life quality, a declined poverty level, the strengthening of sanitation and hygiene, and antibiotics awareness programs which have occurred at a grassroots level across the country in recent years.

On the other hand, the subgroup analysis of adult and child combined vs. only child in our SRMA revealed the increased susceptibility of children to *Shigella* spp. infection in comparison to adults. The highest prevalence of mecillinam resistance was found to be 93.7% (95% CI: 89.7–97.7%). The rate of ciprofloxacin in children was found to be 62.3% (95% CI: 64.0–107.9%), which was more elevated than that of Iran at 3% (95% CI:1–6%) (5). The rate of ceftriaxone resistance at 32.4% (95% CI: 30.0–35.1%) also follows the same pattern as that of Iran at 28% (95% CI: 10–49%). However, a declining rate was observed in the case of trimethoprim–sulfamethoxazole at 74% (95% CI: 65.0–84.3%) and ampicillin at 43.0% (95% CI: 25.6–72.4%), whereas the resistance rate of trimethoprim–sulfamethoxazole and ampicillin was found to be 84% (95% CI: 73–93%) and 69% (95% CI: 56–80%), respectively, in Iran [50]. Ciprofloxacin is used as the first line of treatment for shigellosis for all ages presenting with bloody diarrhea, whereas ceftriaxone and azithromycin are considered for the second line of treatment [51]. Therefore, if this increased rate of ciprofloxacin and ceftriaxone resistance among children in Bangladesh continues to be ignored, it may bring devastating consequences in the near future and contribute to fatal outcomes.

A key strength of this meta-analysis is that this is the first ever compilation of antibiotic-resistant *Shigella* spp. prevalence in Bangladesh. This meta-analysis was carried out with a significant number of studies with a remarkable number of participants. We analyzed all the data and interpreted all of our findings in an unbiased manner. There is no previous study which has had an impactful effect in terms of changing the pooled prevalence of any-drug- and multi-drug-resistant *Shigella* spp., thereby strengthening the robustness and trustworthiness of our study.

Several limitations of our meta-analysis are to be noted, which demands a cautious interpretation of our results. First, this study did not incorporate all of the districts of Bangladesh, hence the estimated results may not truly reflect the magnitude of antibiotic-resistant *Shigella* spp. strains in Bangladesh. No assessment was conducted on the possible impact of the age, sex, ethnicity, socioeconomic status, and lifestyle of the patients on the prevalence of antibiotics resistance because these types of data were unavailable. We could also not analyze extensively drug resistance (XDR) and poly-drug resistance (PDR) due to limited data. Only one previous study, conducted by Rahman et al. in 2017 [28], reported XDR, but no information was found on PDR in any of the included studies. In addition, this study integrated information using different methods. The degree of variation should be limited though as the majority of the studies used the disk diffusion method and complied to the guidelines of the CLSI. Lastly, because of the unavailability of prior antibiotic usage and hospitalization records in our included studies, we could not incorporate these data into our meta-analysis. The possible risk factors for MDR *Shigella*, the severity, and the outcome were also not addressed comprehensively because of partial data availability.

To our knowledge, this is the first study to comprehensively assess the overall prevalence of commonly used antibiotics resistant against *Shigella* spp. in Bangladesh. As our findings revealed that resistance to the first choice of antibiotics, such as fluoroquinolones and ciprofloxacin, against *Shigella* spp. was significantly higher at a rate of 61.9% and 31.1%, respectively, it is alarming that these antibiotics might cease to be effective very soon and will thus no longer be first-choice antibiotics. As plasmid-mediated quinolones-resistant genes (qnr A, qnr B, qnr S, and aac (6′)-Ib-cr) as well as mutations in the genes (gyrA and parC) of quinolone-resistance-determining regions (QRDRs) can lead to fluoroquinolones, including ciprofloxacin resistance [52,53,54], it is likely that horizontal gene transfer will make other species resistant. Due to the lack of new antibiotics, it is crucial to use the ones that are already available with caution. There is evidence that the appropriate and moderate use of antibiotic can reduce resistance [55]. Since irrational antibiotic usage is extensive in Bangladesh through prescription and self-medication, this can be accomplished by enacting tougher laws on antibiotic use, as well as by educating healthcare professionals and the community [56,57,58]. Antibiotic stewardship programs can also help to mitigate the abuse of antibiotics. Additionally, standardization of the monitoring process is crucial in order to acquire more comparable and high-quality data. Meanwhile, there needs to be regular and nationwide surveillance in order to keep an eye on the patterns of resistance of the pathogens. This will help healthcare professionals to make informed decisions about the appropriate treatment options for their patients. Furthermore, it can aid in the development of new and more effective antibiotics.

## 4. Materials and Methods

### 4.1. Guideline and Protocol

The PRISMA guidelines were followed to conduct this systematic review and meta-analysis (SRMA) [59]. The study’s protocol was submitted to PROSPERO for registration (CRD42023407828).

### 4.2. Literature Search Strategy and Study Selection

To determine relevant studies on the prevalence of antimicrobial-resistant *Shigella* spp. in Bangladesh, a thorough literature search was conducted up until 11 March 2023. The search included PubMed, Web of Science, Scopus, and Google Scholar databases. Using the appropriate search phrases and keywords listed in Table S1, all qualifying articles were obtained from the four databases. Moreover, EndNote X9 software (Clarivate Analytics, London, UK) was used to import the reference lists of the selected studies and checked for the elimination of any duplicated studies.

Eventually, the remaining publications were chosen for an examination of their abstracts after the elimination of redundant studies. Three authors (SA, MIHC, and SS) independently reviewed the titles, abstracts, and subsequent complete texts of the articles to determine their eligibility. Finally, discussions among the authors were performed to settle any disagreements regarding a study’s inclusion.

### 4.3. Eligibility Criteria

Studies were only considered eligible for inclusion in this systematic review and meta-analysis if they provided enough information to estimate the prevalence of antimicrobial resistance in shigellosis patients from Bangladesh, independent of their gender, race, or age. Studies with only abstracts, case reports, review articles, theses, editorials, and studies without any information on *Shigella* spp. and their antibiotic susceptibility were not included in the analysis. Furthermore, unpublished articles or studies with insufficient information as well as non-human studies were also excluded from this study. However, no limitations were imposed on the publication year and language.

### 4.4. Definitions and Data Abstraction

Any-drug resistance (DR): resistance to any antibiotics used to treat shigellosis. Multi-drug resistance (MDR): resistance to at least three classes of antibiotics [60]. Mono-drug resistance (Mono DR): resistance to only a single antibiotic.

Three authors (SA, MIHC, and SS) independently extracted data from each of the eligible studies, and any discrepancies were reviewed by the fourth author (MAI). The following data were then gathered and organized into a spreadsheet: first author’s last name, year of publication, study design, study location, length of patient enrolment, patient count, method/medium used to test for antibiotic susceptibility, participants’ ages, sex of the patients, name of the tested antibiotics, and prevalence of DR.

### 4.5. Quality Assessment

Based on the Joanna Briggs Institute (JBI) critical appraisal tools for prevalence studies [61], two authors (SSA and MAI) independently evaluated the quality of each included study. The other authors confirmed the assessment results further, and any significant inconsistencies were addressed through discussion. When the overall score was <50%, 50–70%, or >70%, studies were classified as having a “high risk of bias” (a poor quality), “moderate risk of bias,” (a moderate quality), or “low risk of bias” (a high quality), respectively [62,63].

### 4.6. Data Analysis

The pooled prevalence was calculated with 95% confidence intervals (CIs) using the random-effects model. While calculating the heterogeneity, the *I*^2^ statistic and Cochran’s Q tests were used. An *I*^2^ value of >75% indicated substantial heterogeneity, and *p* < 0.05 was regarded as statistically significant after that. In order to investigate publication bias, the prevalence estimate was plotted against the sample variance in a funnel plot. When at least 10 papers were available, Egger’s test was used to validate the funnel plot’s asymmetry. The Stata 14.0 (Statistical Software, College Station, TX, USA, 2015), metaprop codes in the meta (version 6.1-0) and metafor (version 3.8-1) packages of R (version 4.2.2) in RStudio (version 1.2.5033) were used to create all analyses and visualizations [64].

### 4.7. Subgroup and Sensitivity Analysis

The prevalence of antibiotic resistance between adults and children combined vs. only children was estimated in the subgroup analysis. Sensitivity analyses were carried out based on the following methodologies: by omitting (1) small (*n* < 100) and (2) low- and moderate-quality studies in order to determine the cause of heterogeneity and checking the robustness of the results (high risk of bias).

## 5. Conclusions

This meta-analysis revealed a higher resistance to commonly used antibiotics as well as the emergence of higher multi-drug resistance against *Shigella* spp. Therefore, the setting up of an antimicrobial surveillance system and the implementation of preventative measures is required, such as educating the public about the effects of indiscriminate antibiotic use and prohibiting the sale of antibiotics without a prescription to tackle the therapeutic challenges of shigellosis.

## Figures and Tables

**Table 1:** Major characteristics of the included studies.

Study ID [References]	Study Design	Study Area	Study Duration	Total Number of Tested Samples	Age Groups	AST Method	Tested Antibiotics
Huq 2023 [23]	Cross-sectional	Kushtia	2016 to 2018	12	All ages <5 years; 5 to 18 years; ≥18 years	Disk diffusion method	AMP, AMC, TET, STM, KAN, CIP, FEP, NAL, CRO, and CHL
Pholwat 2022 [24]	Cohort study	Dhaka	January 2019 to September 2019	154	NR	Broth microdilution	AMP, AZM, CIP, and T-S
Nuzhat 2022 [25]	Cross-sectional	Dhaka and Matlab	January 2001 to December 2020	2146	<5 years	Disk diffusion method	CIP, MEC, AZM, and CTX
Garbern 2021 [21]	Cross-sectional	Dhaka	March 2019 to March 2020	42	Children (<18 years); adult (≥18 years)	Disk diffusion method	CEP, AMG, FQ, MAC, PEN, TET, and T-S
Houpt 2020 [26]	Cohort study	Dhaka	January 2019 to September 2019	149	All ages (5 months–58 years)	Disk diffusion method	CIP, T-S, AMP, AMC/CALV, CHL, TET, CRO, and MEM
Gruninger 2017 [27]	Cross-sectional	Dhaka	January 2009 to December 2014	230	Children (>5 years of age)	Disk diffusion method	CIP
Rahman 2017 [28]	Cross-sectional	Dhaka	June 2015 to December 2015	134	NR	Disk diffusion method	AMP, T-S, CIP, AZM, MEC, CRO/CFM, and MEM
Shahunja 2020 [22]	Cross-sectional	Dhaka	June 2014 to May 2017	7	Children (>5 years of age)	Disk diffusion method	AMP, CIP, AZM, CRO, CFM, and AMK
Iqbal 2014 [29]	Cross-sectional	Dhaka	2006 to 2011	200	NR	Disk diffusion method	AMP, AZM, CRO, CHL, CIP, NAL, SUL, TMP, NOR, STM, TET, MEL, GEN, KAN, and AMK
Das 2013 [20]	Cross-sectional	Dhaka	2000 to 2012	2960	NR	Disk diffusion	TMP, SUL, AMP, NAL, MEC, and CIP
Uddin 2013 [30]	Cross-sectional	Dhaka	2001 to 2011	200	All ages	Disk diffusion method	AMP, STM, TET, CIP, NAL, MEC, T-S, CRO, CTX, CAZ, and IPM
Ahmed 2012 [31]	Cross-sectional	Dhaka	January 2005 to December 2008	2847	NR	Disk diffusion method	AMP, CRO, CHL, CIP, T-S, ER, NAL, and TET
Rahman 2007 [32]	Cross-sectional	Dhaka	2001 to 2002	266	NR	Disk diffusion method	AMP, CHL, T-S, TET, NAL, CIP, ME, CRO, and GEN
Talukder 2006 (a) [33]	Cross-sectional	Dhaka	January 1999 to December 2004	113	NR	Disk diffusion method	AMP, MEC, NAL, T-S, and CIP
Talukder 2006 (b) [34]	Cross-sectional	Dhaka	January 1999 and December 2003	445	NR	Disk diffusion method	AMP, TET, MEC, NAL, T-S, AZM, CIP, NOR, OFX, and CRO
Khan 2004 [35]	Cross-sectional	Dhaka	January 1997 to December 2001	227	Children	Disk diffusion method	AMP, T-S, TET, CIP, NAL, and MEC
Talukder 2003 (a) [36]	Cross-sectional	Dhaka	January 1997 to June 2001	358	NR	Disk diffusion method	AMP, CIP, NAL, and MEC
Talukder 2003 (b) [37]	Cross-sectional	Dhaka	January 2000 to September 2002	144	NR	Disk diffusion method	AMP, T-S, TET, CIP, NAL, and MEC
Talukder 2002 [38]	Cross-sectional	Dhaka	January 1999 to December 2002	21	NR	Disk diffusion method	AMP, T-S, CIP, NAL, and MEC
Hossain 1998 [39]	Cross-sectional	Dhaka (urban) and Matlab (rural	January 1991 to December 1996	14,915	NR	Disk diffusion method	AMP, T-S, TET, CIP, NAL, and MEC
Mamun 1997 [40]	Cross-sectional	Rajbari	January 1995 to December 1995	63	All ages (4 months to 65 years)	Controlled diffusion method and disk diffusion method	AMP, T-S, TET, CIP, NAL, and CHL
Jahan 1997 [41]	Cross-sectional	Rajbari	January 1994 to June 1995	21	All ages (0 months to >20 years)	Disk diffusion method	AMP, T-S, TET, CIP, NAL, and CHL
Chowdhury 1995 [42]	Cross-sectional	Matlab	1991 to 1992	721	NR	Disk diffusion method	AMP, T-S, PIV, and NAL
Dhar 1992 [43]	Cross-sectional	Dhaka	NR	23	NR	Disk diffusion method	AMK, AMP, CHL, CEF, GEN, NAL, T-S, TMP, and TET
Bennish 1992 [44]	Cross-sectional	Dhaka, Matlab, and Mirzapur	1983 to 1990	16,344	NR	Disk diffusion method	AMP, T-S, and NAL
Munshi 1987 [45]	Cross-sectional	Teknaf	January to April 1985, January to December 1986, January to February 1987	1179	NR	Disk diffusion method	SUL, NAL, TET, CHL, AMP, TMP, and STM
Tacket 1984 [46]	Cross-sectional	Dhaka	January to October and December 1982	136	NR	Disk diffusion method	AMP, CHL, GEN, KAN, STM, TET, and T-S
Stoll 1982 [3]	Cross-sectional	Dhaka	December 1979 to November 1980	412	All ages (<1 to ≥60 years)	Disk diffusion method	AMP, CHL, GEN, KAN, and TET

AST: antibiotic susceptibility testing; AMP: ampicillin; T-S: trimethoprim–sulfamethoxazole; NAL: nalidixic acid; CIP: ciprofloxacin, MEC: mecillinam, STM: streptomycin, TET: tetracycline, KAN: Kanamycin, CRO: ceftriaxone, AMC/CALV: amoxicillin/clavulanate, MEM: meropenem, PEN: penicillin, FQ: fluoroquinolones, CHL: chloramphenicol, PIV: pivmecillinam, CEF: cephalothin, GEN: gentamicin, TMP: trimethoprim, SUL: sulphathiazole and AMK: amikacin, CFM: cefixime, ER: erythromycin, NOR: norfloxacin, OFX: ofloxacin, CTX: cefotaxime, CAZ: ceftazidime, IMP: imipenem, CEP: cephalosporin, AMG: aminoglycoside, MAC: macrolide, FEP: cefepime, AMC: amoxicillin; NR: not reported.

**Table 2:** Sensitivity analyses.

Antibiotics	Prevalence of Antibiotic Resistance [95% CIs] (%)	Difference in Pooled Prevalence Compared to the Main Result	Number of Studies Analyzed	Total Number of Shigellosis Samples
**Any-DR [excluding small studies (*n* < 100)]**				
Kanamycin	0.3 [0.2–0.3]	2.9% lower	1	396
Cefixime	1.9 [1.7–2.1]	Unchanged	1	266
Chloramphenicol	3.9 [0.2–64.3]	7.7% lower	3	811
Gentamicin	4.1 [3.7–4.7]	Unchanged	1	266
Amoxicillin/clavulanate	10.7 [9.1–12.6]	Unchanged	1	149
Ceftriaxone	11.3 [3.4–37.3]	0.5% higher	5	1350
Mecillinam	13.7 [5.5–34.1]	Unchanged	6	20,485
Pivmecillinam	19.3 [17.9–20.7]	Unchanged	1	721
Nalidixic	31.3 [11.1–88.4]	4.9% lower	13	35,440
Ampicillin	33.2 [22.8–48.3]	1.3% lower	16	35,167
Azithromycin	35.2 [16.3–75.9]	3.6% lower	3	1003
Ciprofloxacin	39.5 [13.0–119.7]	8.4% higher	9	8917
Tetracycline	46.3 [19.8–108.3]	4.1% lower	7	1832
Erythromycin	58.0 [50.5–66.6]	Unchanged	1	200
Streptomycin	65.0 [53.4–79.1]	31.2% higher	1	100
Trimethoprim–sulfamethoxazole	67.8 [57.9–79.4]	7% higher	14	3679
**Any-DR (excluding low- and moderate-quality studies)**				
Kanamycin	0.3 [0.2–0.3]	2.9% lower	1	396
Aminoglycoside	1.2 [0.9–1.6]	Unchanged	1	42
Cephalosporin	7.1 [5.3–9.7]	Unchanged	1	42
Chloramphenicol	15.0 [0.3–809.3]	3.4% higher	3	480
Mecillinam	19.3 [6.2–60.1]	5.6% higher	4	20,085
Ciprofloxacin	20.2 [5.5–73.9]	10.9% lower	7	8313
Macrolide	26.2 [19.4–35.4]	Unchanged	1	42
Ampicillin	27.4 [16.8–44.6]	7.1% lower	10	32,728
Nalidixic acid	29.2 [7.1–120.0]	7.0% lower	8	32,242
Ceftriaxone	32.4 [30.0–35.1]	21.6% higher	1	601
Tetracycline	33.0 [13.1–83.0]	17.4% lower	5	1099
Azithromycin	37.5 [21.5–65.3]	1.3% lower	2	610
Penicillin	40.5 [29.9–54.8]	Unchanged	1	42
Fluoroquinolones	61.9 [45.7–83.8]	Unchanged	1	42
Trimethoprim–sulfamethoxazole	68.1 [55.2–84.0]	7.3% higher	10	21,440
**Multi-DR [excluding small (*n* < 100) and low- and moderate-quality studies]**				
Excluding small studies	32.8 [13.7–78.6]	0.6% lower	6	4037
Excluding low- and moderate-quality studies	50.4 [24.9–102.1]	17% higher	4	3223

CIs: Confidence intervals.

## Data Availability

All data relevant to this review are included in the text, Supplementary Materials, and references.

## Supplementary Materials

The following supporting information can be downloaded at: https://www.mdpi.com/article/10.3390/antibiotics12050817/s1, Figure S1: Subgroup analysis estimating the prevalence of any-drug resistance against *Shigella* spp. in adult and children vs. children only; Table S1: Search strategy; Table S2: Quality assessment of cross-sectional studies using the Joanna Briggs Institute critical; Table S3: Quality assessment of cohort studies using the Joanna Briggs Institute critical.
