# Supplementary material for: Prevalence of Antibiotic-Resistant Shigella spp. in Bangladesh: A Systematic Review and Meta-Analysis of 44,519 Samples
Source: Antibiotics (Basel). 2023 Apr 26;12(5):817. doi: 10.3390/antibiotics12050817 (PMC10215428; doi:10.3390/antibiotics12050817)
Supplement: Supplementary file 1 [file antibiotics-12-00817-s001.zip › Figure_S1_Subgroup_Adult+Children Vs children only.pdf]

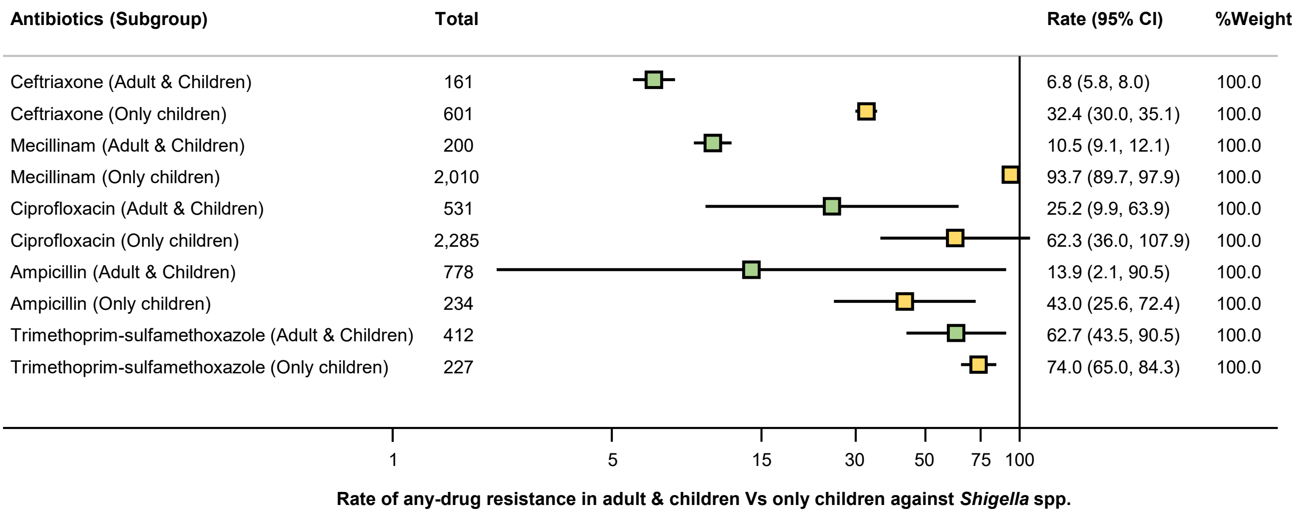

Supplementary Figure S1. Subgroup analysis estimating the prevalence of any-drug resistance against *Shigella* spp. in adult and children vs children only.
