# Supplementary material for: Prevalence of Antibiotic-Resistant Shigella spp. in Bangladesh: A Systematic Review and Meta-Analysis of 44,519 Samples
Source: Antibiotics (Basel). 2023 Apr 26;12(5):817. doi: 10.3390/antibiotics12050817 (PMC10215428; doi:10.3390/antibiotics12050817)
Supplement: Supplementary file 1 [file antibiotics-12-00817-s001.zip › Table S1_Search strategies.pdf]

Table S1. Search Strategy

Databases	Search Strategies
**PubMed**	((Shigella[Title/Abstract] OR Shigellosis[Title/Abstract] OR "Bacillary dysentery"[Title/Abstract] OR "Bloody diarrhea"[Title/Abstract] OR diarrhea[Title/Abstract] OR diarrhoea[Title/Abstract]) AND (Bangladesh[Title/Abstract] OR Dhaka[Title/Abstract] OR Chittagong[Title/Abstract] OR Chattogram[Title/Abstract] OR Rajshahi[Title/Abstract] OR Rangpur[Title/Abstract] OR Barisal[Title/Abstract] OR Barishal[Title/Abstract] OR Sylhet[Title/Abstract] OR Khulna[Title/Abstract] OR Mymensingh[Title/Abstract] OR Dinajpur[Title/Abstract] OR Bogra[Title/Abstract] OR Comilla[Title/Abstract] OR Cumilla[Title/Abstract] OR Faridpur[Title/Abstract] OR Pabna[Title/Abstract] OR Noakhali[Title/Abstract] OR "Cox's Bazar"[Title/Abstract] OR Jessore[Title/Abstract] OR Jashore[Title/Abstract] OR Satkhira[Title/Abstract] OR Gazipur[Title/Abstract] OR Kushtia[Title/Abstract] OR Sirajganj[Title/Abstract] OR Gopalganj[Title/Abstract] OR Jamalpur[Title/Abstract] OR Tangail[Title/Abstract] OR Manikganj[Title/Abstract] OR Patuakhali[Title/Abstract] OR Rangamati[Title/Abstract] OR Chandpur[Title/Abstract] OR Netrakona[Title/Abstract] OR Magura[Title/Abstract] OR Naogaon[Title/Abstract] OR Nilphamari[Title/Abstract])) AND (isoniazid[Title/Abstract] OR rifampin[Title/Abstract] OR rifampicin[Title/Abstract] OR rifamycin[Title/Abstract] OR ethambutol[Title/Abstract] OR pyrazinamide[Title/Abstract] OR streptomycin[Title/Abstract] OR amikacin[Title/Abstract] OR kanamycin[Title/Abstract] OR capreomycin[Title/Abstract] OR viomycin[Title/Abstract] OR enviomycin[Title/Abstract] OR Ciprofloxacin[Title/Abstract] OR Levofloxacin[Title/Abstract] OR Moxifloxacin[Title/Abstract] OR ethionamide[Title/Abstract] OR prothionamide[Title/Abstract] OR seromycin[Title/Abstract] OR Terizidone[Title/Abstract] OR Rifabutin[Title/Abstract] OR clarithromycin[Title/Abstract] OR Linezolid[Title/Abstract] OR thioacetazone[Title/Abstract] OR Bedaquiline[Title/Abstract] OR Clofazimine[Title/Abstract] OR rifapentine[Title/Abstract] OR cephalosporin[Title/Abstract] OR ceftazidime[Title/Abstract] OR ceftriaxone[Title/Abstract] OR cefepime[Title/Abstract] OR beta lactam[Title/Abstract] OR Extended spectrum beta lactam[Title/Abstract] OR penicillin[Title/Abstract] OR nalidixic acid[Title/Abstract] OR resistance*[Title/Abstract] OR resistant*[Title/Abstract] OR susceptibilit*[Title/Abstract] OR sensitivit*[Title/Abstract])
**Web of Science**	TI=(Shigella OR Shigellosis OR “Bacillary dysentery” OR “Bloody diarrhea” OR diarrhea OR diarrhoea) AND TI=(Bangladesh OR Dhaka OR Chittagong OR Chattogram OR Rajshahi OR Rangpur OR Barisal OR Barishal OR Sylhet OR Khulna OR Mymensingh OR Dinajpur OR Bogra OR Comilla OR Cumilla OR Faridpur OR Pabna OR Noakhali OR "Cox's Bazar" OR Jessore OR Jashore OR Satkhira OR Gazipur OR Kushtia OR Sirajganj OR Gopalganj OR Jamalpur OR Gazipur OR Tangail OR Manikganj OR Patuakhali OR Rangamati OR Chandpur OR Netrakona OR Magura OR Naogaon OR Nilphamari) AND TI=(isoniazid OR rifampin OR rifampicin OR rifamycin OR ethambutol OR pyrazinamide OR streptomycin OR amikacin OR kanamycin OR capreomycin OR viomycin OR

	enviomycin OR Ciprofloxacin OR Levofloxacin OR Moxifloxacin OR ethionamide OR prothionamide OR seromycin OR Terizidone OR Rifabutin OR clarithromycin OR Linezolid OR thioacetazone OR Bedaquiline OR Clofazimine OR rifapentine OR cephalosporin OR ceftazidime OR ceftriaxone OR cefepime OR “beta lactam” OR penicillin OR nalidixic acid OR resistance* OR resistant* OR susceptibilit* OR sensitivit*)
**Scopus**	TITLE-ABS (shigella OR shigellosis OR "Bacillary dysentery" OR "Bloody diarrhea" OR diarrhea OR diarrhoea) AND TITLE-ABS (bangladesh OR dhaka OR chittagong OR chattogram OR rajshahi OR rangpur OR barishal OR barisal OR sylhet OR khulna OR mymensingh OR dinajpur OR bogra OR comilla OR cumilla OR faridpur OR pabna OR noakhali OR "Cox's Bazar" OR jessore OR jashore OR satkhira OR gazipur OR kushtia OR sirajganj OR gopalganj OR jamalpur OR gazipur OR tangail OR manikganj OR patuakhali OR rangamati OR chandpur OR netrakona OR magura OR naogaon OR nilphamari) AND TITLE-ABS (isoniazid OR rifampin OR rifampicin OR rifamycin OR ethambutol OR pyrazinamide OR streptomycin OR amikacin OR kanamycin OR capreomycin OR viomycin OR enviomycin OR ciprofloxacin OR levofloxacin OR moxifloxacin OR ethionamide OR prothionamide OR seromycin OR terizidone OR rifabutin OR clarithromycin OR linezolid OR thioacetazone OR bedaquiline OR clofazimine OR rifapentine OR cephalosporin OR ceftazidime OR ceftriaxone OR cefepime OR "beta lactam" OR penicillin OR "nalidixic acid" OR resistance OR resistant OR susceptibility OR sensitivity)
**Google Scholar**	allintitle:(Shigella OR Shigellosis OR “Bacillary dysentery” OR “Bloody diarrhea”) Bangladesh
