# Supplementary material for: Prevalence of Antibiotic-Resistant Shigella spp. in Bangladesh: A Systematic Review and Meta-Analysis of 44,519 Samples
Source: Antibiotics (Basel). 2023 Apr 26;12(5):817. doi: 10.3390/antibiotics12050817 (PMC10215428; doi:10.3390/antibiotics12050817)
Supplement: Supplementary file 1 [file antibiotics-12-00817-s001.zip › Table S2_Quality assessment_Cross-sectional.pdf]

Table S2. Quality assessment of cross-sectional studies using the Joanna Briggs Institute critical appraisal tool

Study ID	Q1	Q2	Q3	Q4	Q5	Q6	Q7	Q8	Yes (%)
Huq 2023									57.1
Nuzhat 2022									71.4
Garbern 2021									100.0
Gruninger 2017									100.0
Rahman 2017									57.1
Shahunja 2020									100.0
Iqbal 2014									57.1
Das 2013									71.4
Uddin 2013									71.4
Ahmed 2012									71.4
Rahman 2007									71.4
Talukder 2006(a)									57.1
Talukder 2006(b)									57.1
Khan 2004									71.4
Talukder 2003									57.1
Talukder 2002(a)									57.1
Talukder 2002(b)									57.1
Hossain 1998									71.4
Mamun 1997									71.4
Jahan 1997									71.4
Chowdhury 1995									57.1

**Dhar 1992**	No	Yes	Yes	Yes	No	Not applicable	Yes	No	57.1
**Bennish 1992**	No	Yes	Yes	Yes	No	Not applicable	Yes	Yes	71.4
**Munshi 1987**	No	Yes	Yes	Yes	No	Not applicable	Yes	No	57.1
**Tacket 1984**	No	Yes	Yes	Yes	No	Not applicable	Yes	No	57.1
**Stoll 1982**	No	Yes	Yes	Yes	No	Not applicable	Yes	Yes	71.4

Yes No Not applicable

Q1: Were the criteria for inclusion in the sample clearly defined?
Q2: Were the study subjects and the setting described in detail?
Q3: Was the exposure measured in a valid and reliable way?
Q4: Were objective, standard criteria used for measurement of the condition?
Q5: Were confounding factors identified?
Q6: Were strategies to deal with confounding factors stated?
Q7: Were the outcomes measured in a valid and reliable way?
Q8: Was appropriate statistical analysis used?
