# Supplementary material for: Prevalence of Antibiotic-Resistant Shigella spp. in Bangladesh: A Systematic Review and Meta-Analysis of 44,519 Samples
Source: Antibiotics (Basel). 2023 Apr 26;12(5):817. doi: 10.3390/antibiotics12050817 (PMC10215428; doi:10.3390/antibiotics12050817)
Supplement: Supplementary file 1 [file antibiotics-12-00817-s001.zip › Table S3_Quality assessment_Cohort.pdf]

Table S3. Quality assessment of cohort studies using the Joanna Briggs Institute critical appraisal tool

Study ID	Q1	Q2	Q3	Q4	Q5	Q6	Q7	Q8	Q9	Q10	Q11	Yes (%)
Pholwat 2022	Unclear	Unclear	Yes	No	Not applicable	Yes	Yes	Unclear	No	No	Yes	40.0
Houpt 2020	Unclear	Unclear	Yes	No	Not applicable	Yes	Yes	Yes	Yes	Not applicable	Yes	66.7

Yes **No** **Unclear** **Not applicable**

Q1: Were the two groups similar and recruited from the same population?
Q2: Were the exposures measured similarly to assign people to both exposed and unexposed groups?
Q3: Was the exposure measured in a valid and reliable way?
Q4: Were confounding factors identified?
Q5: Were strategies to deal with confounding factors stated?
Q6: Were the groups/participants free of the outcome at the start of the study (or at the moment of exposure)?
Q7: Were the outcomes measured in a valid and reliable way?
Q8: Was the follow up time reported and sufficient to be long enough for outcomes to occur?
Q9: Was follow up complete, and if not, were the reasons to loss to follow up described and explored?
Q10: Were strategies to address incomplete follow up utilized?
Q11: Was appropriate statistical analysis used?
